# Group V Phospholipase A_2_ Mediates Endothelial Dysfunction and Acute Lung Injury Caused by Methicillin-Resistant *Staphylococcus Aureus*

**DOI:** 10.3390/cells10071731

**Published:** 2021-07-08

**Authors:** Yu Maw Htwe, Huashan Wang, Patrick Belvitch, Lucille Meliton, Mounica Bandela, Eleftheria Letsiou, Steven M. Dudek

**Affiliations:** Division of Pulmonary, Critical Care, Sleep and Allergy, Department of Medicine, University of Illinois at Chicago, Chicago, IL 60612, USA; yhtwe2@uic.edu (Y.M.H.); hswang@uic.edu (H.W.); pbelvitc@uic.edu (P.B.); lmeliton@uic.edu (L.M.); mbande4@uic.edu (M.B.); eletsiou@uic.edu (E.L.)

**Keywords:** gVPLA_2_, sPLA_2_-V, PLA_2_G5, secretory phospholipase A_2_, endothelium, ALI, bacterial infection, intravital microscopy, ARDS, MRSA, USA300

## Abstract

Lung endothelial dysfunction is a key feature of acute lung injury (ALI) and clinical acute respiratory distress syndrome (ARDS). Previous studies have identified the lipid-generating enzyme, group V phospholipase A2 (gVPLA_2_), as a mediator of lung endothelial barrier disruption and inflammation. The current study aimed to determine the role of gVPLA_2_ in mediating lung endothelial responses to methicillin-resistant *Staphylococcus aureus* (MRSA, USA300 strain), a major cause of ALI/ARDS. In vitro studies assessed the effects of gVPLA_2_ inhibition on lung endothelial cell (EC) permeability after exposure to heat-killed (HK) MRSA. In vivo studies assessed the effects of intratracheal live or HK-MRSA on multiple indices of ALI in wild-type (WT) and gVPLA_2_-deficient (KO) mice. In vitro, HK-MRSA increased gVPLA_2_ expression and permeability in human lung EC. Inhibition of gVPLA_2_ with either the PLA2 inhibitor, LY311727, or with a specific monoclonal antibody, attenuated the barrier disruption caused by HK-MRSA. LY311727 also reduced HK-MRSA-induced permeability in mouse lung EC isolated from WT but not gVPLA_2_-KO mice. In vivo, live MRSA caused significantly less ALI in gVPLA_2_ KO mice compared to WT, findings confirmed by intravital microscopy assessment in HK-MRSA-treated mice. After targeted delivery of gVPLA_2_ plasmid to lung endothelium using ACE antibody-conjugated liposomes, MRSA-induced ALI was significantly increased in gVPLA_2_-KO mice, indicating that lung endothelial expression of gVPLA_2_ is critical in vivo. In summary, these results demonstrate an important role for gVPLA_2_ in mediating MRSA-induced lung EC permeability and ALI. Thus, gVPLA_2_ may represent a novel therapeutic target in ALI/ARDS caused by bacterial infection.

## 1. Introduction

Acute respiratory distress syndrome (ARDS) is a severe inflammatory disorder of the lungs which causes high mortality (up to 45%) and significant lifelong medical challenges in survivors [1,2]. Although advanced ventilator management and supportive therapies have improved mortality over time, no effective pharmacologic therapy is available that targets the underlying acute lung injury (ALI) process [1,3,4]. Pneumonia and sepsis are the most common causes of ARDS [1,2], with antibiotic-resistant organisms frequently being involved [5]. *Staphylococcus aureus* bacteria are gram-positive, coagulase-positive members of the Staphylococcaceae family [6]. The first penicillin-resistant *S. aureus* strain emerged in 1942, with methicillin-resistant strains (MRSA) identified several years later in 1960 [6]. MRSA infections are difficult to treat due to their resistance to various antibiotics and are now a major burden on the health care system [6,7]. Meta-analyses indicate that MRSA-induced sepsis results in longer hospital stay, increased utilization of hospital resources, and increased costs [8,9].

The secretory phospholipase A_2_ (sPLA_2_) family of proteins are enzymes that hydrolyze phospholipids at the sn-2 position to release free fatty acids and lysophospholipids [10,11]. The sPLA_2_ enzymes produce multiple lipid mediators, such as leukotrienes that promote inflammation and modulate immune responses [12,13]. At least eleven different mammalian sPLA_2_ enzymes have been identified to date, with several implicated in the pathogenesis of ALI/ARDS in both animal models and patients [14,15,16,17,18]. Of these, gVPLA_2_ (or sPLA_2_-V) is a 14-kDa enzyme that mediates multiple biological effects including lipid metabolism, eicosanoid production, leukocyte migration, airway inflammation, transcriptional activity, phagocytosis, and thrombosis [13,19,20,21,22]. 

Prior studies have suggested that gVPLA_2_ may play a central role in ALI pathogenesis by mediating lung injury through multiple independent mechanisms. It disrupts pulmonary surfactant within the alveoli, a key step in ALI development [2], by binding surfactant phosphatidylcholine with high affinity, resulting in its hydrolysis and damage [23]. Transgenic mice overexpressing gVPLA_2_ die in the neonatal period due to lung surfactant dysfunction and diffuse alveolar damage similar to ARDS, while transgenic mice overexpressing the related inflammatory enzyme gXPLA_2_ survive with normal surfactant composition [24]. Other studies have implicated gVPLA_2_ in mediating lung endothelial barrier dysfunction and leukocyte recruitment, which are major characteristics of ALI [2,25]. GVPLA_2_ increases pulmonary endothelial cell (EC) permeability through direct hydrolysis of the cell membrane and participates in the inflammatory responses associated with LPS- or mechanical stretch-induced EC dysfunction [26,27,28,29]. Inhibition of gVPLA_2_ attenuates polymorphonuclear leukocyte activation and migration [22] and reduces cellular leukotriene synthesis [30,31]. Prior work by our group and others has also demonstrated that inhibition or genetic deletion of gVPLA_2_ attenuates murine ALI induced by LPS or high tidal volume ventilation [28,29,32]. These prior studies provide a strong scientific premise for the important role of gVPLA_2_ in ARDS [18]. The current study seeks to characterize the role of gVPLA_2_ in mediating lung EC dysfunction and ALI induced by the clinical-relevant pathogen MRSA (USA300 strain).

## 2. Materials and Methods

*Reagents:* LY311727, an sPLA_2_ inhibitor, and MCL-3G1 (mAb directed against gVPLA_2_) were purchased from Cayman Chemical (Ann Arbor, MI, USA). The plasmid for gVPLA_2_ (PLA2G5-pCMV6-Entry vector) was purchased from OriGene (Rockville, MD, USA). Mouse angiotensin-converting enzyme (ACE) antibody was obtained from R&D Systems (Minneapolis, MN, USA). All other reagents were obtained from MilliporeSigma (St. Louis, MO, USA), unless otherwise noted. 

*Human lung endothelial cell culture:* Human pulmonary artery endothelial cells (HPAEC) and human lung microvascular endothelial cells (HLMVEC) were obtained from Lonza (Walkersville, MD, USA) and cultured according to the manufacturer’s instructions as previously described [26]. Endothelial cells (EC) were grown in endothelial growth medium-2 (EGM-2) at 37 °C in a 5% CO_2_ incubator. Passages 5–7 were used for experiments.

*Mouse pulmonary vascular endothelial cell culture:* Mouse pulmonary vascular EC (mPVEC) were isolated from gVPLA_2_ knockout (gene designation *Pla2g5*) or wild-type (C57Bl/6) mice, as we have described [33,34]. Flow cytometry was used to sort the mPVEC through the selection of CD31+/CD45- cells [33]. mPVECs (passages 13–19) were grown in endothelial growth medium-2 (EGM-2) at 37 °C in a 5% CO_2_ incubator.

*Preparation of live and heat-killed MRSA:* The USA300 CA-MRSA wild-type (LAC) strain used in this study was kindly provided by Dr. Jiwang Chen (UIC). Bacteria were cultivated as described previously [35]. To prepare the heat-killed (HK-MRSA) bacterial solutions used in some experiments, live MRSA suspension was placed in 60 °C water bath for one hour. The HK-MRSA was diluted in PBS, aliquoted, and stored at −80 °C until the day of the experiment.

*Transendothelial electrical resistance assay (TER/ECIS):* EC monolayer barrier function was assessed using the electric cell-substrate impedance sensing system (ECIS) (Applied Biophysics, Troy, NY, USA), as we have described previously [26,36]. Briefly, ECs grown in wells containing gold film electrodes were grown to confluence and then treated in various experiments with vehicle (DMSO), LY 311727 (100 µM), or MCL-3G1 (25 μg/mL) for 1 h followed by HK-MRSA (1–2 × 10^8^ CFU/mL). Transendothelial electrical resistance (TER) values were measured over time. Values are expressed as mean normalized resistance compared to baseline values, with the means of several wells pooled in each independent experiment. 

*XPerT permeability assay:* Interendothelial cell gaps were quantified using the XPerT assay, as described before [37,38]. Briefly, confluent HLMVECs grown on biotinylated gelatin coated plates were pre-treated with vehicle (DMSO) or LY 311727 (100 µM) for 1 h, followed by HK-MRSA (2 × 10^8^ CFU/mL) challenge. Eight hours later, FITC-conjugated avidin (Invitrogen) was added to the media, and after washing, images were immediately obtained with a Nikon TE2000-S microscope (Nikon, Tokyo, Japan) at 10 × magnification. Corresponding brightfield images are provided for each condition in Supplementary Figure S2. Four to five random fields were imaged for each condition. Gap formation is defined by green fluorescence signal (matrix bound FITC-avidin) and ImageJ 1.48v (National Institutes of Health, Bethesda, MD, USA) was used to quantify the area of green immunofluorescence, as we have described previously [38,39].

*Mouse model of MRSA(USA300)-induced acute lung injury:* All experimental conditions and animal care procedures were approved by the University of Illinois at Chicago (UIC) Animal Care and Use Committee. Eight to twelve week-old male homozygous gVPLA_2_ knockout (gVPLA_2_ KO) [29,40] and C57BL/6 wild type (WT) mice were anesthetized (ketamine/xylazine) and then administrated live MRSA intratracheally (IT) (0.75 × 10^8^ CFU per mouse (25 gr)) or an equal volume of PBS (vehicle). For a specific set of experiments, gVPLA_2_ KO mice were administered liposomes containing gVPLA_2_ plasmid or empty vector (10 mg/kg, 100 μL) by intravenous (IV) retro-orbital injection 24 h before MRSA treatment (described in more detail below). Eighteen hours after MRSA, mice were euthanized, and samples were collected as we have described before (bronchoalveolar lavage (BAL) and lung tissue) [41]. BAL was first centrifuged to separate cells and the BAL fluid, and then total protein levels in BAL fluid were measured with the BCA protein assay kit (ThermoFisher Scientific, Waltham, MA, USA). Total BAL cell counts were determined using the Bio-Rad TC10 automated cell counter device. Differential BAL cell counts were assessed after staining of cytospin slides with Kwik Diff Stain (ThermoFisher Scientific, Waltham, MA, USA). Lung tissues were harvested and processed for histology (hematoxylin and eosin (H&E) staining) as described [41]. Lung histology was performed at the Research Histology and Tissue Imaging Core of UIC. Histology slides were scanned using a Leica Aperio AT2 at 40×. Images were analyzed using Aperio ImageScope (Leica Biosystems, Buffalo Grove, IL, USA). Lung injury score was calculated as described before [42].

*ACE antibody-conjugated liposome delivery of gVPLA_2_ plasmid:* Angiotensin converting enzyme (ACE) antibody was conjugated to liposomes to target the pulmonary vasculature selectively, as previously described [43]. ACE antibody was crosslinked to liposomes containing gVPLA_2_ plasmid (gVPLA_2_-lipos) or empty vector (Ctr-lipos). The liposomes were prepared under sterile conditions and administrated to mice retrorbitally as a 100-μL aliquot of sterile ACE-conjugated liposomes (containing 10 mg/kg gVPLA_2_ or control plasmid).

*Immunoblotting analysis:* In vitro samples: HPAEC were cultured to confluence and then the medium was replaced with fresh basal medium. EC were stimulated with either vehicle control or HK-MRSA (2 × 10^8^ CFU/mL) at different time points. EC were subsequently washed with cold Ca2+/Mg2+-free PBS and lysed with cell lysis buffer (Cell Signaling) containing protease and phosphatase inhibitors. Equal protein EC lysates were then mixed with 6× SDS-sample buffer (Boston BioProducts, Ashland, MA, USA) and boiled. In vivo samples: Equal volumes of BAL fluid samples were mixed with 6× SDS sample buffer and boiled before electrophoresis. Sample proteins were separated with handcast 15% SDS-PAGE gels and transferred onto nitrocellulose membranes (Bio-Rad, Hercules, CA, USA). Membranes were then immunoblotted with anti-gVPLA_2_ primary antibody (MCL-3G1) (4 °C, overnight) followed by secondary anti-mouse antibody conjugated to HRP (room temperature, 1 h). Protein expression was detected with enhanced chemiluminescence (Pierce ECL, Rockford, IL, USA) on HyBlot CL film (Thomas Scientific, Swedesboro, NJ, USA). Blots were scanned and quantitatively analyzed using ImageJ software.

*2-Photon intravital microscopy:* WT or gVPLA_2_ KO male mice were anesthetized (ketamine/xylazine) and injected with 2 × 10^8^ CFU HK-MRSA per mouse (25 gr) intratracheally (IT). Control mice received an equal volume of PBS. After 18 h, mice were anesthetized, intubated and connected to a mechanical ventilator (Harvard Apparatus, Inspira Ventilator) at the following settings: tidal volume 10 mL/kg, respiratory rate 100 breaths/min, and PEEP 0 cm H2O. Mice were then injected (IV) with tetramethylrhodamine (TMR)-conjugated dextran (70 kDa, 20 mL/kg) and Alexa488-conjugated Gr1 antibody (0.1 mg/kg), to label plasma leak and neutrophils respectively. A left thoracotomy was then performed to expose the lung surface. Next, a custom made “lung window” equipped with glass coverslip and vacuum pressure was positioned over the exposed lung and suction applied until the tissue was stabilized against the coverslip, as described elsewhere [44]. Image acquisition: Time series images between 30 s and 5 min were obtained using an Ultima In Vivo Microscopy System (Prairie Technologies) with a 60× Nikon water immersion objective and high-speed resonant scanner. Excitation wavelength was 810 nm. The red emission filter was set to 560–610 nm and the green emission filter was set to 490–540 nm. Between two and five independent fields were imaged per subject. Image processing and data analysis: ImageJ software was used for processing and analysis. Raw data time series in .TIF format stacks containing images throughout the respiratory cycle were first processed by a custom-made macro which enabled the isolation of images from the same point in different respiratory cycles. Images at end inspiration (as defined by maximal alveolar size/airspace area) were chosen for analysis. Channels containing data from red and green emission filters were analyzed separately and later merged for presentation. Green channel (490–540 nm) images were passed through a median filter to reduce background and isolate signal only from grouped pixels which defined individual neutrophils. Only neutrophils observed over consecutive respiratory cycles were quantified per imaging field and averaged for each subject. Red channel images (560–610) were used to identify the interstitial area of each field. The threshold function was used to quantify the total rhodamine labeled area per field and was expressed as a ratio of the interstitial to total image area. This parameter was then averaged over 2–5 fields per subject.

*Statistical analysis:* Values in graph bars are expressed as means ± standard deviation (SD). Values in ECIS graphs are expressed as means ± standard error (SE). Statistical analyses were conducted with GraphPad Prism 8 software. Comparisons between groups were made using *t*-test (for comparisons between two groups only) or analysis of variance (ANOVA) followed by Tukey’s post-hoc test (for comparisons of more than two groups). *p* values less than <0.05 were considered statistically significant.

## 3. Results

### 3.1. MRSA Induces EC Barrier Dysfunction and Increases the Expression of gVPLA_2_

**Figure 1:**
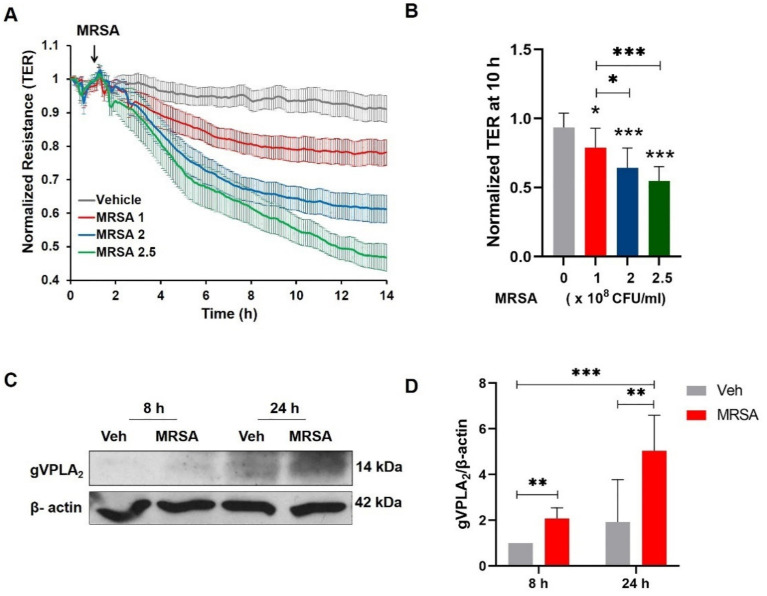
MRSA induces lung endothelial barrier disruption and up-regulates gVPLA_2_ expression. (**A**, **B**): HPAEC monolayers cultured on ECIS arrays were challenged with three different concentrations of HK-MRSA (1, 2, or 2.5 × 10^8^ CFU/mL). (**A**): Transendothelial electrical resistance (TER) values were recorded over time. (**B**): Normalized TER values at the 10-h time point. * *p* < 0.05, *** *p* < 0.001. All conditions were significant vs. control. Other comparisons are indicated by brackets. *n* = 8–17/condition. (**C**, **D**): HPAEC were stimulated with HK-MRSA (2 × 10^8^ CFU/mL) for 8 or 24 h. gVPLA_2_ expression was assessed in cell lysates by immunoblotting. (**C**): Representative blots of gVPLA_2_ and β-actin. (**D**): Densitometry analysis of gVPLA_2_ normalized to β-actin. ** *p* < 0.01, *** *p* < 0.001. *n* = 5.

Lung endothelial barrier disruption is a critical early step in ARDS pathogenesis [45]. In the current study, we first evaluated the effects of heat-killed MRSA USA300 (HK-MRSA) on lung EC barrier function using ECIS, a highly sensitive method for obtaining real-time permeability data [26,36]. HK-MRSA significantly reduced EC resistance in a concentration- (1–2.5 × 10^8^ CFU/mL) and time-dependent manner (Figure 1A,B), indicating that the EC barrier integrity had been disrupted. Prior studies have demonstrated that gVPLA_2_ expression is increased in EC after stimulation with barrier disrupting agents such as LPS and mechanical stretch [26,28]. Therefore, we next assessed the effects of MRSA on gVPLA_2_ expression in lung EC. A significant increase in gVPLA_2_ protein expression was observed in lung EC after 8 or 24 h treatment with HK-MRSA (Figure 1C,D).

### 3.2. Inhibition of gVPLA_2_ Attenuates MRSA-Induced Lung EC Permeability

**Figure 2:**
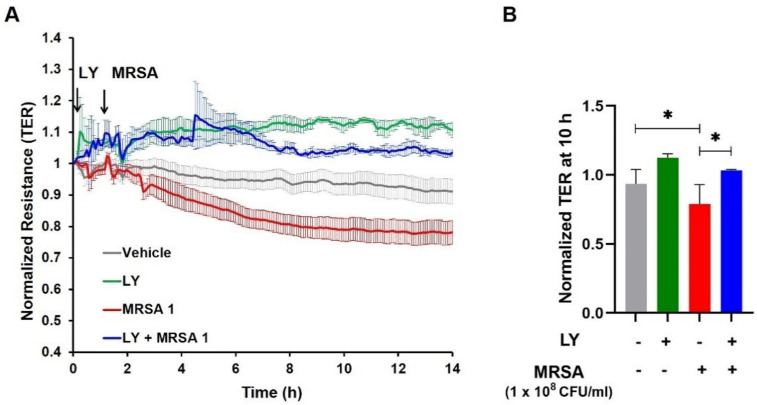
sPLA_2_ inhibitor, LY311727, attenuates HK-MRSA-induced lung endothelial barrier disruption. HPAEC were pre-treated with LY311727 (100 µM, 1 h) and challenged with HK-MRSA (1 × 10^8^ CFU/mL). (**A**): Transendothelial electrical resistance (TER) values were recorded over time. (**B**): Normalized TER values at the 10-h time point. * *p* < 0.05. *n* = 3–16/condition.

Next, we evaluated the functional role of gVPLA_2_ in MRSA-induced EC barrier dysfunction using multiple complementary modalities: pharmacological inhibition, specific blocking antibody, and lung EC derived from genetically-deficient gVPLA_2_ mice. LY311727, a potent global sPLA_2_ inhibitor [46], was first used. For these experiments, HPAEC were treated with LY311727 (100 µM) for 1 h, followed by HK-MRSA (1–2 × 10^8^ CFU/mL). LY311727 elevated baseline TER as early as 15 min after treatment, which persisted throughout the duration of the experiment, although this effect was not statistically significant. Notably, LY311727 completely reversed the reduction in TER induced by the lower concentration of HK-MRSA (1 × 10^8^ CFU/mL) (Figure 2A,B), and significantly attenuated TER reduction by the higher concentration of HK-MRSA (2 × 10^8^ CFU/mL) (Supplementary Figure S1A,B).

**Figure 3:**
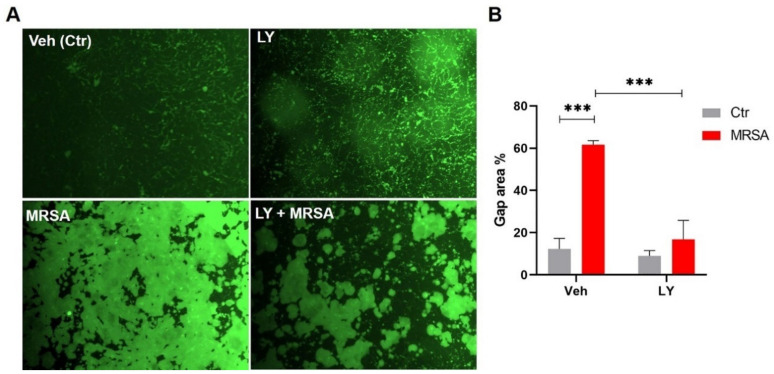
sPLA_2_ inhibitor, LY311727, attenuates HK-MRSA-induced lung endothelial intercellular gap formation. HLMVEC monolayers were grown to confluency on biotinylated gelatin coated plates. Cells were pretreated with LY311727 (100 µM, 1 h) and then challenged with HK-MRSA (2 × 10^8^ CFU/mL) for 8 h. FITC-avidin was added to media and allowed to permeate cells to reach biotin substrate at site of paracellular gaps (indicated by green fluorescence). Images were taken using a fluorescence microscope. (**A**): Representative immunofluorescence pictures of interendothelial cellular gaps (10× magnification). (**B**): Bar graph represents gap area (FITC) quantification. *** *p* < 0.001. *n* = 3.

To characterize additional properties of EC barrier function in MRSA-induced dysfunction, intercellular gap formation was assessed by measuring permeability to FITC-avidin as described in the Methods section. This assay provides unique information about local monolayer barrier function [37] by allowing for visualization and quantification of the gaps between individual cells. For these experiments, we used HLMVEC, which form tighter barriers than HPAEC and have fewer gaps at baseline [39]. HK-MRSA (2 × 10^8^ CFU/mL, 8 h) significantly increased the gap area in HLMVEC compared to control (62% vs. 12%, *p* < 0.001) (Figure 3A,B). However, the sPLA_2_ inhibitor, LY311727, significantly attenuated the MRSA-induced formation of intracellular gaps (Figure 3A,B). Corresponding brightfield images are provided for each condition in Supplementary Figure S2.

**Figure 4:**
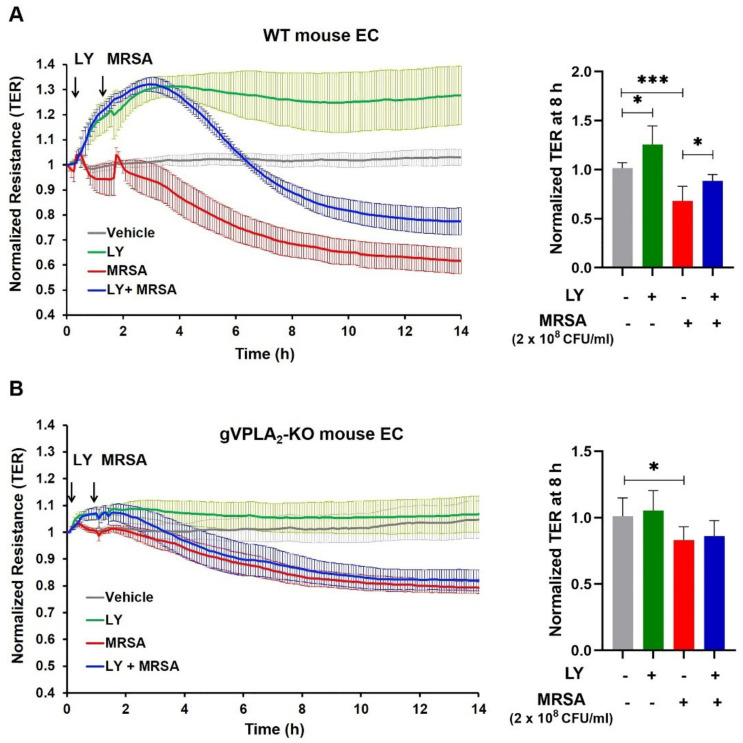
Effects of sPLA_2_ inhibitor, LY311727, on HK-MRSA-induced EC barrier disruption in wild-type and gVPLA_2_-deficient mPVEC. (**A**): Wild-type (WT) or (**B**): gVPLA_2_-deficient (KO) mPVEC (isolated murine pulmonary endothelial cells) were pretreated with LY311727 (100 µM, 1 h) and then challenged with HK-MRSA (2 × 10^8^ CFU/mL). Depicted are pooled ECIS tracings (left panels) and normalized TER values at 8-h time point (right panels). * *p* < 0.05, *** *p* < 0.001. *n* = 4–11/condition.

To examine whether LY311727 protects against HK-MRSA-induced EC permeability by targeting the gVPLA_2_ isoform specifically, we next employed murine lung EC (mPVEC) isolated from gVPLA_2_ (Pla2g5) global knockout mice in comparison to EC isolated from WT mice as controls [34]. In WT mPVEC, HK-MRSA caused a significant decrease in TER over time as expected (Figure 4A), while sPLA_2_ inhibition with LY311727 elevated baseline TER and attenuated the MRSA-induced EC permeability, similar to the effects observed in human lung EC (Figure 2A,B). In contrast, LY311727 had no effect on MRSA-induced TER reduction in gVPLA_2_-deficient mPVEC, suggesting an important role for gVPLA_2_ in mediating MRSA effects (Figure 4B). Moreover, we observed that in WT mPVEC, MRSA caused a 33% reduction in TER at the 8-h time point (compared to Veh), while in gVPLA_2_ KO mPVEC TER were reduced by only 18%, further supporting a role for gVPLA_2_ in mediating EC permeability (Figure 4A,B, right panels).

**Figure 5:**
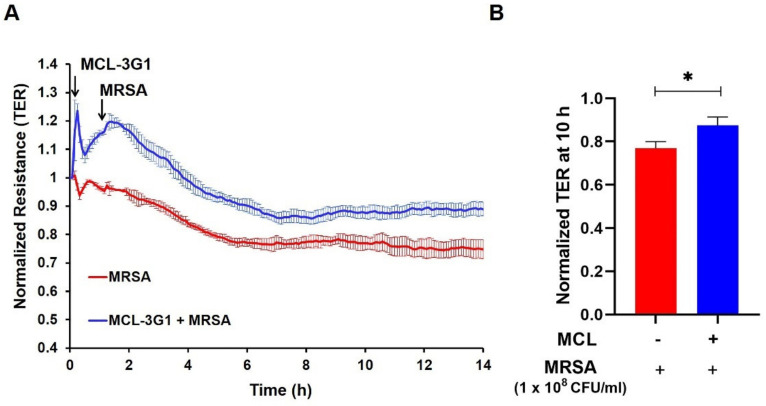
HK-MRSA-induced lung endothelial barrier dysfunction is attenuated by a monoclonal blocking antibody against gVPLA_2_ (MCL-3G1). HPAEC were pre-treated with MCL-3G1 (25 μg/mL, 1 h) and then challenged with HK-MRSA (1 × 10^8^ CFU/mL). (**A**): TER values were recorded over time. (**B**): Normalized TER values at the 10-h time point. * *p* < 0.05. *n* = 3–4/condition.

We then investigated the effects of specific targeting of gVPLA_2_ in HK-MRSA-challenged human lung EC. For these experiments, HPAEC were treated with a monoclonal antibody specifically directed against the gVPLA_2_ enzyme (MCL-3G1) [47]. This antibody inhibits EC barrier disruption induced by LPS [26]. In the current experiments, HK-MRSA-induced TER reduction was attenuated by pretreatment with MCL-3G1, suggesting a protective role of MCL-3G1 against MRSA-induced EC barrier disruption (Figure 5A,B).

### 3.3. gVPLA_2_ Deficiency Protects against ALI In Vivo

**Figure 6:**
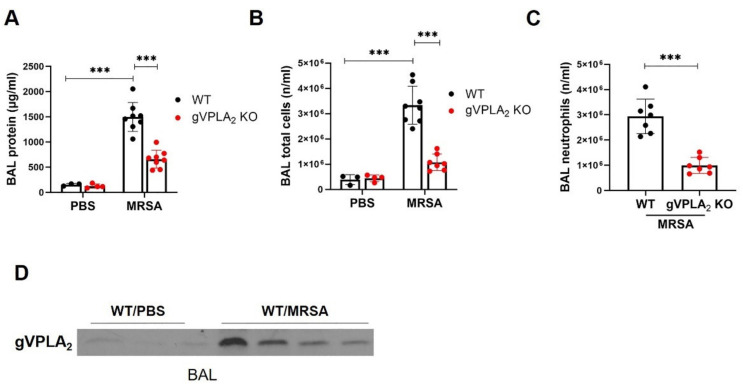
gVPLA_2_-deficient mice are protected from MRSA-induced lung injury. C57BL/6 (WT) and gVPLA_2_ knockout (gVPLA_2_ KO) mice were treated with live MRSA-USA300 (0.75 × 10^8^ CFU/mouse) or PBS intratracheally. After 18 h, (**A**) total protein, (**B**) total cell counts, and (**C**) neutrophil counts were assessed in the bronchoalveolar lavage (BAL). (**D**): gVPLA_2_ levels in BAL fluid were assessed by immunoblotting. *** *p* < 0.001. *n* = 3–8 mice/condition.

To further evaluate the role of gVPLA_2_ in the pathogenesis of MRSA-induced ALI, in vivo experiments were performed in gVPLA_2_-deficient mice (gVPLA_2_ KO). At 18 h after intratracheal administration of live MRSA (USA300), we observed a dramatic increase in BAL protein levels in WT mice compared to controls (1388 ± 234 vs. 154.7 ± 24 μg/mL), indicative of increased lung vascular leakage, which is a critical ALI feature (Figure 6A). This MRSA-induced hyperpermeability was attenuated in gVPLA_2_ KO mice, with the BAL protein levels significantly decreased by 52% compared to WT mice (Figure 6A). Another characteristic of ALI is the accumulation of inflammatory immune cells in the alveolar space. MRSA caused a significant increase in the number of total cells in BAL, while in gVPLA_2_ KO mice this accumulation was dramatically reduced by 65% (Figure 6B). Differential cell analysis of the BAL cells revealed that the majority of recruited cells after MRSA were neutrophils (90–95%), but neutrophil infiltration was significantly reduced in gVPLA_2_-deficient mice compared to WT (Figure 6C). BAL neutrophil levels were very low in non-MRSA treated mice (data not shown). In addition, increased levels of gVPLA_2_ protein were observed in BAL fluid from WT mice infected with MRSA compared to non-infected animal controls (Figure 6D). 

**Figure 7:**
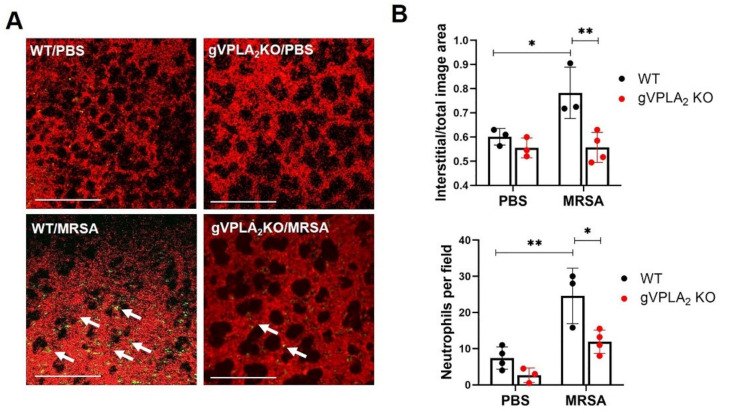
Lung intravital microscopy of gVPLA_2_ KO and WT mice challenged with HK-MRSA. C57BL/6 WT and gVPLA_2_ knockout (gVPLA_2_ KO) mice were challenged with HK-MRSA-USA300 (2 × 10^8^ CFU/mouse, IT) or PBS and imaged by intravital microscopy 18 h later. (**A**): Representative images show the lung interstitial area labeled with TMR-dextran (red) and fluorescent-labeled neutrophils (green), also indicated by white arrows. Scale bar: 200 μm. (**B**): Quantification of the interstitial area and neutrophil accumulation. * *p* < 0.05 ** *p* < 0.01. *n* = 3–4 mice/condition.

Next, we employed intravital microscopy to image biological processes in the lung tissue of living mice in real time [48]. This in vivo imaging technique was used to visualize how gVPLA_2_ expression modulates neutrophil recruitment and interstitial edema in the lungs during MRSA-induced ALI. For these experiments, HK-MRSA was used instead of live bacteria because of biosafety concerns during the imaging process. Before imaging, mice were injected with TMR-dextran to stain the vasculature, and a fluorescent antibody (Alexa488-Gr1) was infused to label leukocytes. In WT mice, HK-MRSA caused dramatic recruitment of neutrophils into the lungs, extravasation of dextran tracer into the alveolar space, an indicator of vascular leakage, and interstitial thickening (Figure 7). However, lung imaging in gVPLA_2_ KO mice demonstrated a significant decrease in neutrophil recruitment and plasma extravasation into the airspaces after HK-MRSA challenged compared to WT (Figure 7).

**Figure 8:**
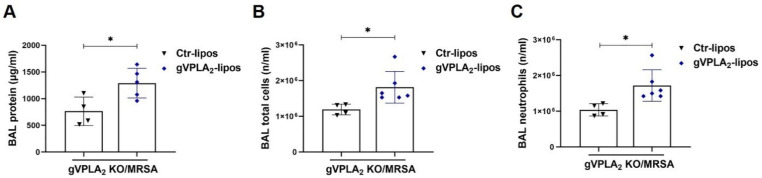
Exacerbation of MRSA-induced lung injury in gVPLA_2_ KO mice by administration of ACE antibody-conjugated liposomes carrying gVPLA_2_ plasmid. gVPLA_2_ knockout (gVPLA_2_ KO) mice were administrated intravenously ACE-conjugated liposomes containing either gVPLA_2_ plasmid (gVPLA_2_-lipos) or empty vector (Ctr-lipos). Twenty-four hours later, mice were treated with live MRSA-USA300 (0.75 × 10^8^ CFU/mouse) intratracheally. (**A**): total protein levels, (**B**) total cell counts, and (**C**) neutrophil counts were determined in the bronchoalveolar lavage (BAL) of mice 18 h after MRSA treatment. * *p* < 0.05. *n* = 4–6 mice/condition.

Our data have demonstrated that gVPLA_2_ plays a major role in regulating MRSA-induced EC permeability in vitro, and that mice globally deficient in gVPLA_2_ are protected against MRSA-induced ALI in vivo. To assess the specific functional role of lung endothelial gVPLA_2_ in vivo, we next performed “gain of function” studies in gVPLA_2_ KO mice using ACE-antibody linked liposomes (ACE-lipos) to target delivery of gVPLA_2_ plasmid to the pulmonary endothelium. As described previously, these liposomes deliver their cargo selectively to the lung vasculature [43]. For these experiments, gVPLA_2_ KO mice were administrated ACE-liposomes containing gVPLA_2_ or control plasmid before pulmonary infection with MRSA. As shown in Figure 8, gVPLA_2_ KO mice that received gVPLA_2_-ACE-liposomes have increased total protein levels in BAL, and exhibit significantly elevated accumulation of neutrophils in the alveolar space, compared to gVPLA_2_ KO mice that received control-ACE-liposomes. These data demonstrate that expression of gVPLA_2_ in the lung vasculature contributes to MRSA-induced ALI pathogenesis. 

**Figure 9:**
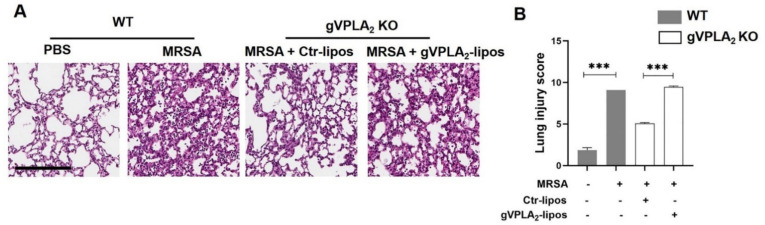
Effects of gVPLA_2_ expression on lung tissue histopathology. H&E staining of lung sections that were obtained from WT or gVPLA_2_ KO mice treated with live MRSA-USA300 (0.75 × 10^8^ CFU/mouse) intratracheally for 18 h. gVPLA_2_ KO mice were administrated intravenously ACE-conjugated liposomes containing either gVPLA_2_ plasmid (gVPLA_2_-lipos) or empty vector (Ctr-lipos). Lung tissues were scanned at 40× and representative pictures were taken at 20×. (**A**): Shown are representative images from each experimental group. Scale bar: 200 μm. (**B**): Graph bars depict lung injury scores for each experimental group. *** *p* < 0.001. *n* = 3–4 mice/condition.

Analysis of lung histology further supports this interpretation. In the lung tissues of MRSA-challenged mice, several histopathological changes were observed that are characteristic of ALI, including lung edema, pulmonary congestion, inflammatory cell infiltration, and thickening of the alveolar wall. Lung injury scoring was performed on these histologic samples as previously described [42]. These inflammatory changes were ameliorated in gVPLA_2_ KO mice that received control-ACE-liposomes. However, reconstitution of endothelial gVPLA_2_ expression in gVPLA_2_ KO mice by administration of gVPLA_2_-ACE-liposomes resulted in histopathological changes similar to WT mice infected with MRSA (Figure 9). 

## 4. Discussion

**Figure 10:**
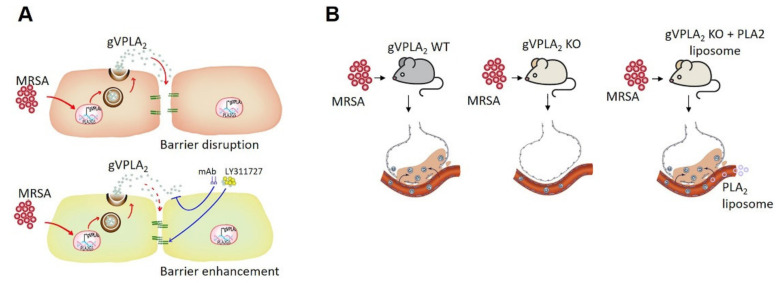
Overview schema of the role of gVPLA_2_ in MRSA-induced ALI. (**A**): Depicted are lung endothelial cells in barrier-disrupted (top) or barrier-enhanced (bottom) conditions. In vitro studies demonstrate that MRSA increases EC permeability and expression of gVPLA_2_. Inhibition of gVPLA_2_ using the inhibitor LY311727 or a monoclonal blocking antibody against gVPLA_2_ (MCL-3G1) attenuates MRSA-induced lung EC permeability and enhances barrier function. (**B**): Depicted are mice and alveolar-capillary units with or without pulmonary edema and neutrophil infiltrates. In vivo studies demonstrate that MRSA-induced lung injury is attenuated in gVPLA_2_ KO mice (white) compared to WT (gray). However, restoration of endothelial gVPLA_2_ expression in gVPLA_2_ KO mice by administration of ACE-conjugated liposomes containing gVPLA_2_ plasmid reverses the protective effects observed in these mice after MRSA.

Data presented in this study support an important role for the lipolytic enzyme gVPLA_2_ in mediating MRSA-induced pulmonary endothelial permeability in vitro and acute lung injury (ALI) in vivo in a preclinical mouse model of ARDS (summarized in Figure 10). First described in 1967 [49], ARDS remains a challenging public health problem despite over 50 years of investigations. ARDS results from either direct lung injury (e.g., bacterial or viral pneumonia) or through indirect mechanisms (e.g., sepsis) [50]. MRSA is a frequent infectious cause of both severe pneumonia and sepsis [51,52] that lead to ARDS. Currently available antibiotics are not always sufficient to control MRSA infection, and emerging drug resistant strains are a challenge in treating MRSA-induced sepsis and ARDS [53], resulting in significant mortality and morbidity [54]. A key step in ARDS development is disruption of lung endothelial barrier function by inflammatory stimuli [25,45]. The primary objective of the current study was to explore the hypothesis that gVPLA_2_ mediates aspects of MRSA-induced lung endothelial dysfunction in vitro and in vivo. For our studies, we employed the CA-MRSA USA300 strain, which is one of the major strains of MRSA currently circulating in the United States [55]. Although USA300 was originally identified as a strain of community-associated MRSA infection, it also occurs in the health care setting and represents a major clinical challenge. Among the virulence-defining characteristics of this specific strain is expression of the gene for the Panton-Valentine leucocidin (PVL) PVL toxin, and other drug resistant elements [56].

In vitro, multiple complementary modalities were used to demonstrate that inhibition of gVPLA_2_ activity or expression reduces permeability caused by MRSA in cultured lung EC. These modalities included the pharmacological inhibitor LY311727 (Figure 2, Figure 3 and Figure 4), the specific blocking antibody MCL-3G1 (Figure 5), and lung EC derived from genetically-deficient gVPLA_2_ mice (Figure 4). LY311727 is a small molecule sPLA_2_ inhibitor that significantly attenuated HK-MRSA-induced EC permeability as measured by ECIS and quantification of intercellular gap formation. MRSA-induced EC barrier dysfunction also was attenuated in mPVEC isolated from gVPLA_2_ KO mice (i.e., these EC are completely deficient in gVPLA_2_) compared to WT mPVEC. Moreover, LY311727 inhibited MRSA-induced barrier dysfunction in WT-mPVEC but had no significant effects in the KO mPVEC, suggesting that its protective properties against MRSA-induced permeability require gVPLA_2_ expression. The MCL-3G1 monoclonal Ab against gVPLA_2_ also significantly attenuated MRSA effects on EC barrier function, which further supports the primary observation that this increased permeability is mediated by gVPLA_2_. In combination with our prior studies demonstrating that gVPLA_2_ directly causes EC permeability and mediates LPS-induced EC dysfunction [26,27], the current findings provide strong evidence that gVPLA_2_ represents an important regulator of EC barrier responses to ALI-relevant stimuli. In contrast, the related gIIaPLA_2_ enzyme has no effect on EC permeability [26], despite its levels being increased in ARDS [57]. In addition, we are not aware of any published reports implicating other sPLA_2_ isoforms in lung EC permeability, and therefore the current study focused on gVPLA_2_. 

In vivo, intratracheal administration of live MRSA in mice was employed as a clinically-relevant model of ARDS due to direct lung injury. MRSA (USA300 strain) caused significant increases in multiple indices of ALI, including BAL protein, total cell count, and neutrophils (Figure 6), intravital microscopy quantification of lung tissue edema and neutrophil infiltration (Figure 7), and histological lung injury score (Figure 9). Importantly, all these ALI indices were significantly decreased in gVPLA_2_-deficient mice (gVPLA_2_ KO), indicating that gVPLA_2_ expression augments the pathophysiologic effects of MRSA in the lungs. These effects of gVPLA_2_ expression are consistent with prior studies in LPS- and ventilation-induced ALI murine models [28,29]. In addition, in a model of E.coli pneumonia, gVPLA_2_-deficient mice had decreased ICAM-1 and PECAM-1 lung expression levels, and exhibited reduced recruitment of neutrophils into the alveolar space, compared to WT [58]. In vitro, gVPLA_2_ mediates mechanical stretch-induced ICAM-1 expression, inflammatory cytokine release and neutrophil adhesion to lung EC [28]. Excessive accumulation of neutrophils into the lungs is an important contributor to ALI progression [59], and our study in combination with these prior studies strongly support a role for gVPLA_2_ in regulating this neutrophil accumulation during ALI. 

Although studies using global gVPLA_2_ KO mice provide important insights, gVPLA_2_ is expressed in several cell types relevant to ALI, including lung endothelium, epithelium, and macrophages [20]. Thus, the primary source of gVPLA_2_ responsible for mediating MRSA effects is uncertain from these global knockdown studies. To address the hypothesis that lung EC expression of gVPLA_2_ is functionally important in vivo, we employed ACE-antibody linked liposomes (ACE-lipos) [43] to target delivery of gVPLA_2_ plasmid selectively to the pulmonary endothelium of gVPLA_2_ KO mice. In these experiments, selective expression of gVPLA_2_ in lung EC significantly increased MRSA-induced ALI in gVPLA_2_ KO mice (Figure 8 and Figure 9). The results of these “gain of function” studies support a critical role for lung endothelial gVPLA_2_ in mediating MRSA-induced pulmonary injury. 

To our knowledge, this is the first study to establish a functional role for gVPLA_2_ in the MRSA-induced pathophysiology that occurs during ALI/ARDS. One key step by which MRSA causes injury may involve the upregulation of gVPLA_2_ expression. Prior clinical observations have suggested a potential association between gVPLA_2_ levels and inflammatory lung injury. The lungs of patients with pneumonia demonstrate increased expression of gVPLA_2_ in lung tissue and alveolar macrophages [60], and gVPLA_2_ has been detected in the BAL of infants with ARDS [61]. In the current study, exposure to MRSA increased gVPLA_2_ protein expression in human lung EC in vitro (Figure 1) and in BAL fluid from mice in vivo (Figure 6). Prior work by our group and others has demonstrated that gVPLA_2_ expression is upregulated in lung EC of various preclinical models of ARDS, including LPS and ventilator-induced lung injury, both in vitro and in vivo [26,28,29]. The mechanism by which MRSA and other inflammatory stimuli increase gVPLA_2_ expression in lung EC is unclear, but we recently reported that gVPLA_2_ degradation in endothelium is mediated by autophagy [34]. Dysregulated autophagy occurs during ALI and other inflammatory pulmonary diseases [62], and thus it is reasonable to speculate that MRSA may alter autophagy in lung EC, leading to increased gVPLA_2_ protein levels due to its decreased degradation (clearance). Alternatively, MRSA might induce gVPLA_2_ expression via increasing mRNA levels as reported for other inflammatory stimuli [26,28]. Given the potent inflammatory role of gVPLA_2_, more studies are needed to characterize how MRSA, as well as other injurious stimuli, regulate its expression. To that end, our ongoing studies suggest that epigenetic mechanisms may contribute to gVPLA_2_ gene expression induced by inflammatory agents, which will be the focus of future work. 

The upregulation of gVPLA_2_ has multiple pro-inflammatory and injurious effects. In the alveolar space, gVPLA_2_ hydrolyzes and degrades critical surfactant phospholipids [24,63]. It also hydrolyzes neutrophils by direct interaction and causes release of fatty acids, lysophospholipids and triggers leukotriene production [31,64]. In addition, gVPLA_2_ activates neutrophils to release VEGF, a potent disruptor of endothelial barrier function [65]. The specific mechanisms by which gVPLA_2_ mediates EC barrier disruption after MRSA exposure remain incompletely defined. However, we previously demonstrated that upregulation of gVPLA_2_ in lung EC results in its increased expression on the cell surface, where it then directly increases permeability through hydrolysis of the EC membrane [27] and stimulates cytoskeletal remodeling, F-actin stress fiber formation, and adherens junction disruption [26]. It is likely that gVPLA_2_ also contributes to the EC cytoskeletal changes that occur during MRSA-induced permeability. Others have reported that *Staph aureus* causes EC permeability through microtubule dysfunction and altered adherens junction interactions [66], and the current study extends these insights into how these bacteria negatively affect EC cytoskeletal structure. Recent reports suggest that gVPLA_2_ binds to receptors or integrins on the plasma membrane, and it also has been detected in the nucleus where it may regulate transcription [19,65]. Therefore, it is also possible that gVPLA_2_ mediates some of its effects via pathways that are independent of its enzymatic activity.

The current study has several limitations. First, HK-MRSA was used for the in vitro experiments, which may not completely recapitulate all the effects of exposure to live bacteria. For example, the MRSA USA300 strain used in this study nearly always contains a gene for the PVL toxin, a major virulence factor. This toxin is inactivated by heating and therefore it remains unknown whether it has an effect on lung EC and gVPLA_2_ signaling. However, the heat-killed model is an established approach for in vitro experiments that has been used in other published studies [66,67]. Our results demonstrate that, even in the absence of toxins, the heat-killed bacteria still induce significant disruption of the lung EC barrier. Moreover, we used both live and heat-killed MRSA for the in vivo experiments and observed that both preparations result in significant lung injury, which is attenuated in mice lacking the *Pla2g5* gene. Secondly, LY311727 is a potent global sPLA_2_ inhibitor that may also affect the activity of other sPLA_2_ enzymes; however, similar results were observed using the gVPLA_2_ specific monoclonal antibody, MCL-3G1, as well as lung EC isolated from genetically-deficient gVPLA_2_ KO mice, strongly supporting a primary role for the gVPLA_2_ enzyme in these effects. Another potential limitation of our study is that we employed only one MRSA strain (CA-MRSA, USA300) in these experiments. Additional studies are needed to determine if other MRSA strains (e.g., HA-MRSA) mediate their injurious effects similarly through gVPLA_2_. Finally, the gVPLA_2_ KO mice are globally deficient in all cell types, and thus some of the protective effects noted in the MRSA ALI model may be due to the lack of gVPLA_2_ in cell types other than lung EC, such as epithelium or macrophages. The ACE-antibody linked liposome experiments support the hypothesis that lung EC expression of gVPLA_2_ plays a primary role in MRSA-induced ALI, but important contributions from other cellular sources are possible.

## 5. Conclusions

In conclusion, this study demonstrates that gVPLA_2_ mediates MRSA(USA-300)-induced EC barrier dysfunction in vitro and ALI in vivo. Inhibition of gVPLA_2_ diminishes MRSA-induced permeability in cultured human lung EC, while gVPLA_2_ KO mice exhibit decreased ALI after exposure to MRSA. Moreover, targeted expression of gVPLA_2_ in the lung EC of gVPLA_2_ KO mice results in augmented MRSA-induced ALI. These novel findings improve our understanding of the pathophysiology of MRSA-induced ARDS and identify gVPLA_2_ as a key inflammatory enzyme in this process. This work suggests that targeting inhibition of gVPLA_2_ may have therapeutic potential in ARDS and supports the pursuit of additional studies in this area.

## Data Availability

The data presented in this study are available on request from the corresponding author.

## Supplementary Materials

The following are available online at https://www.mdpi.com/article/10.3390/cells10071731/s1. Figure S1: sPLA_2_ inhibitor, LY311727, attenuates HK-MRSA-induced lung endothelial barrier disruption, Figure S2: XPerT Assay: Brightfield images of cell monolayers, Figure S3: Unedited blots.
