# Supplementary material for: Group V Phospholipase A2 Mediates Endothelial Dysfunction and Acute Lung Injury Caused by Methicillin-Resistant Staphylococcus Aureus
Source: Cells. 2021 Jul 8;10(7):1731. doi: 10.3390/cells10071731 (PMC8304832; doi:10.3390/cells10071731)
Supplement: Supplementary file 1 [file cells-10-01731-s001.zip › cells-1203880-Supplementary Figure legends-FINAL and figures.pdf]

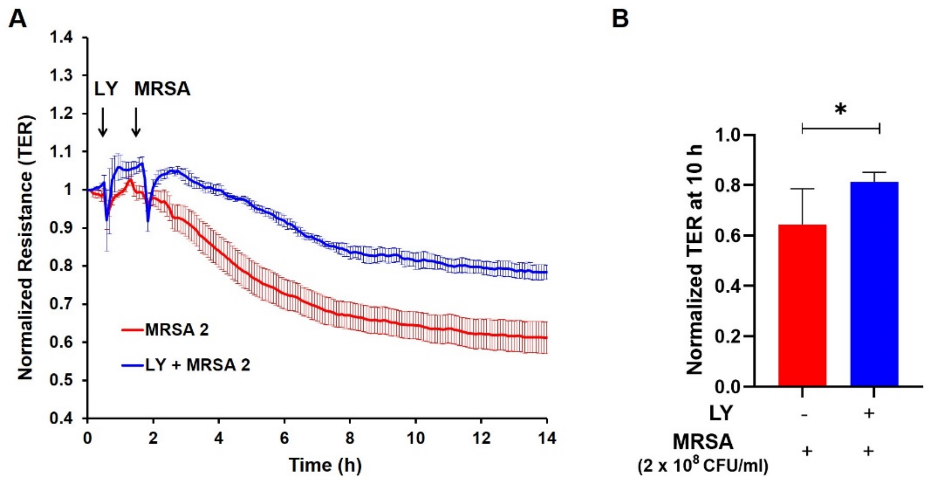

Supplementary Figure S1. **sPLA$_2$ inhibitor, LY311727, attenuates HK-MRSA-induced lung endothelial barrier disruption.** HPAEC were pre-treated with LY311727 (100 μM, 1 hour) and challenged with HK-MRSA (2x 10^8 CFU/ml). (**A**): Transendothelial electrical resistance (TER) values were recorded over time. (**B**): Normalized TER values at the 10-hour time point. *p <0.05. N=4-17/condition.

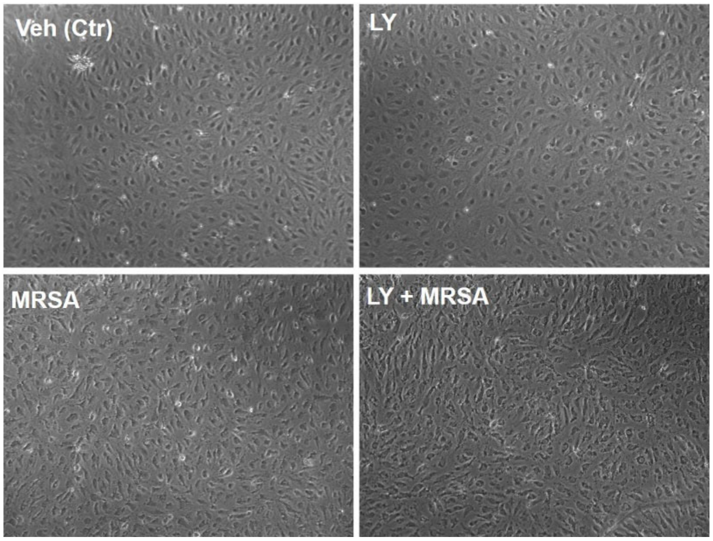

Supplementary Figure S2. **XPerT Assay: Brightfield images of cell monolayers.** HLMVEC monolayers were grown to confluency on biotinylated gelatin coated plates. Cells were pretreated with LY311727 (100 μM, 1 hour) and then challenged with HK-MRSA (2x 10^8 CFU/ml) for 8 hours. FITC-avidin was added to media and allowed to permeate cells to reach biotin substrate at site of paracellular gaps. Images were taken using a fluorescence microscope as shown in Figure 3. Depicted are the corresponding brightfield images (10x).

A

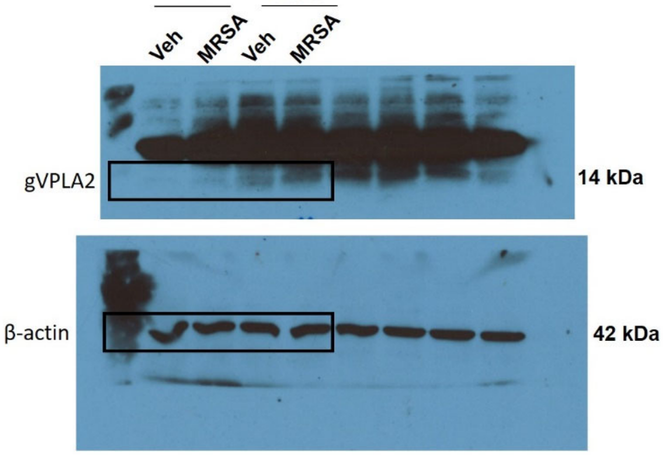

Unedited blots shown in figure 1C

B

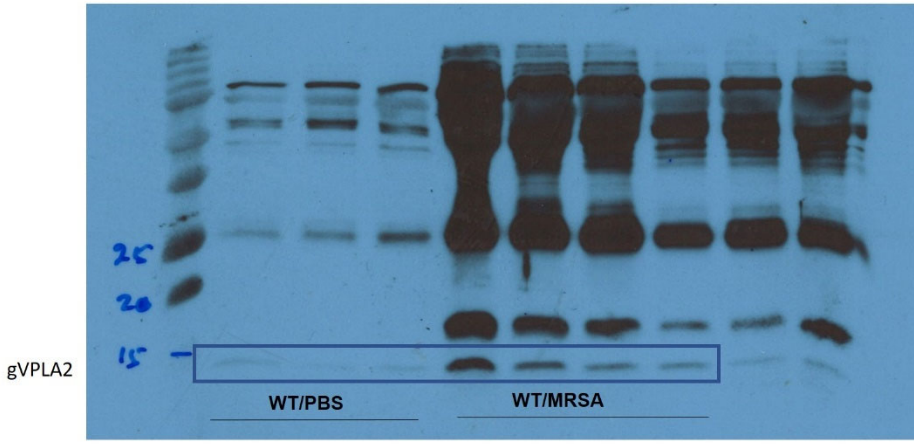

Unedited blot shown in figure 6D

Supplementary Figure S3. Unedited blots for Figure 1C and Figure 6D for shown.
